# Comparison of the Effects of Cyclophosphamide and Mycophenolate Mofetil Treatment Against Immunoglobulin G4-Related Disease: A Retrospective Cohort Study

**DOI:** 10.3389/fmed.2020.00253

**Published:** 2020-07-07

**Authors:** Xuan Luo, Yu Peng, Panpan Zhang, Jieqiong Li, Zheng Liu, Hui Lu, Xuan Zhang, Xiaofeng Zeng, Fengchun Zhang, Yunyun Fei, Wen Zhang

**Affiliations:** ^1^Department of Rheumatology, Peking Union Medical College Hospital, Chinese Academy of Medical Sciences, Peking Union Medical College, Beijing, China; ^2^National Clinical Research Center for Dermatologic and Immunologic Diseases (NCRC-DID), Key Laboratory of Ministry of Health, Beijing, China

**Keywords:** cyclophosphamide, efficacy, IgG4-related disease, mycophenolate mofetil, relapse, response

## Abstract

**Background:** Although there are multiple ways to manage immunoglobulin G4–related disease (IgG4-RD), including treatment with glucocorticoids, “steroid-sparing” immunosuppressive drugs, or biologic agents, few clinical trials on IgG4-RD have been conducted. This study aimed to compare the efficacy and safety of glucocorticoids (GCs) combined with cyclophosphamide (CYC) or mycophenolate mofetil (MMF) in IgG4-RD patients. This cohort study was registered at ClinicalTrials.gov (ID: NCT01670695).

**Methods:** This retrospective study included 155 IgG4-RD patients who received GCs with CYC or MMF at the Department of Rheumatology at Peking Union Medical College Hospital between January 2012 and July 2018. Propensity score matching (PSM) was conducted to match two groups of patients based on their baseline clinical characteristics. Treatment response, relapse rate, and drug safety were analyzed. The treatment response was evaluated based on complete response (CR), partial response (PR), and no change (NC), and the cumulative relapse rate and adverse events in each treatment group were compared using Kaplan–Meier curves and log-rank test, respectively.

**Results:** Of the 155 IgG4-RD patients, 90 were treated with GCs plus CYC (group I) and 65 with GCs plus MMF (group II). After propensity score–matched (PSM) analysis, 108 patients were selected (54 in each group), 49 of whom had “definite” IgG4-RD, 8 “probable” IgG4-RD, and 51 “possible” IgG4-RD. At the last follow-up, the total response in groups I and II was 98.15 and 96.3%, respectively, and within 12 months, the cumulative relapse rate in group II was significantly higher than that in group I (14.8 vs. 3.7%, *P* = 0.046). Recurrence occurred at the paranasal sinus, lacrimal glands, skin, lung, pancreas, and bile ducts, and the relapsed patients achieved remission after switching immunosuppressants or/and increasing the GC dose.

**Conclusions:** In IgG4-RD patients with internal organ involvement, GCs plus CYC or MMF are both effective with similar effects in disease response, while GCs plus CYC reduced the relapse rate better than GCs plus MMF.

## Introduction

Immunoglobulin G4–related disease (IgG4-RD) is a systemic fibroinflammatory condition that affects multiple organs including the pancreas, bile duct, lacrimal gland, salivary gland, thyroid, lung, liver, gastrointestinal tract, kidney, and retroperitoneum (1). It is characterized by tissue infiltration by IgG4-positive cells, and causes prominent pathological changes in most of the affected organs, including lymphoplasmacytic infiltration, storiform fibrosis, and obliterative phlebitis, as well as tumor-like lesions and even depletion (2).

Glucocorticoids (GCs) are the first-line therapy for achieving clinical remission in active or untreated IgG4-RD patients (3). Relapse occurred in about 50% patients with GC tapering or withdrawal (4), and long-term application of GCs may increase the risk of adverse reactions. Thus far, conventional immunosuppressants including azathioprine (AZA) (5), mycophenolate mofetil (MMF), 6-mercaptopurine (6-MP), methotrexate (MTX), tacrolimus, cyclophosphamide (CYC), and leflunomide (LEF) have been used in the treatment of patients with IgG4-RD (6, 7). Data from cohort and randomized controlled trial (RCT) studies show that IgG4-RD can be better controlled with GCs plus conventional immunosuppressants than with GC monotherapy (7–10). However, data comparing the efficacy and safety of combined treatment regimens in IgG4-RD are limited.

MMF, an immunomodulatory agent with antifibrotic effects, can be used for the treatment of IgG4-RD because of its ability to inhibit the transforming growth factor beta pathway (11). CYC is a bifunctional nonspecific cell cycle alkylating agent used for treating tumors, a variety of autoimmune diseases, and IgG4-RD. Our previous studies showed that GCs plus low-dose CYC or MMF can effectively reduce the relapse rate of IgG4-RD (4, 8, 9), but we could not identify which one was more effective in treating IgG4-RD and preventing clinical relapse. Therefore, in this study, we aimed to compare the efficacy of two regimens (GCs plus low-dose CYC or MMF) by retrospectively assessing the response rate, relapse rate, and side effects in eligible IgG4-RD patients who were treated with either of them.

## Methods

This prospective observational cohort study to investigate the disease course and treatment response of IgG4-RD patients was registered on ClinicalTrials.gov (ID: NCT01670695). From January 2012 to July 2018, consecutive patients treated with GCs plus CYC or MMF were retrospectively identified for this study. The study protocol was approved by the ethics board of Peking Union Medical College Hospital, and all study subjects provided written informed consent for study participation.

### Inclusion and Exclusion Criteria

The inclusion criteria were as follows:

1. 18 to 75 years of age.
2. Agreement with the 2011 comprehensive diagnostic criteria for definite, probable, or possible IgG4-RD as follows: (1) characteristic diffuse/localized swelling, sclerosis, or inflammation affecting single or multiple organs; (2) elevated serum IgG4 concentrations (135 mg/dL); (3) increased number of lymphocytes, infiltrated IgG4+ plasma cells (IgG4+ cells/IgG-positive cells >40% and >10 IgG4+ plasma), and fibrosis. Patients who met criteria (1), (2), and (3) were diagnosed with definite IgG4-RD; those who met (1) and (3) were diagnosed with probable IgG4-RD; and those who met (1) and (2) were diagnosed with possible IgG4-RD (12).
3. Presence of internal organ involvement.
4. Use of GC and CYC or MMF for initial treatment and maintenance until the 12-month follow-up.
5. Regularly followed up at months 1, 3, 6, and 12.

The exclusion criteria were as follows:

1. Presenting only with IgG4-related dacryoadenitis and sialadenitis or lymphadenopathy of IgG4-RD.
2. Serious infection.
3. Presence of other rheumatic diseases.
4. Presence of malignant diseases.
5. Women with childbearing potential or currently planning a pregnancy.

### Laboratory Tests, Imaging Studies, and Histological Examination

All patients underwent laboratory tests for complete blood count, erythrocyte sedimentation rate (ESR), hypersensitivity C-reactive protein (hs-CRP) levels, serum immunoglobulin, and IgG subclass concentrations, urinalysis, liver, and renal function tests; and at least one imaging examination, including ultrasonography, digital radiography (DR), computed tomography (CT), magnetic resonance imaging (MRI), or positron emission tomography/computed tomography (PET-CT). In total, 78 patients (72.22%) underwent CT scan, 24 (22.22%) underwent PET scan, 33 (30.56%) underwent MRI, and 49 (45.37%) underwent other imaging examinations, including ultrasound and DR. When necessary, tissue biopsies were analyzed and reviewed by pathologists using previously described methods (12), and laboratory tests and imaging studies were performed during the follow-up period.

### Data Collection

Clinical features, laboratory results, imaging data, adverse events, and details of treatment protocol and disease relapse were evaluated at baseline and at 1, 3, 6, and 12 months after treatment or until relapse.

### Assessment of Disease Activity and Definition of Clinical Response and Relapse

Disease activity per visit was assessed by the IgG4-RD responder index (RI), with a system score of 0–4 for each site/organ system (13). An IgG4-RD RI score ≥3 was used to identify patients with active disease (14).

Three categories of clinical response were defined: (1) complete response (CR), in which IgG4-RD RI <3 and declined ≥2 points after treatment; (2) partial response (PR), in which IgG4-RD RI declined ≥2 points after treatment but still remained ≥3, and if IgG4-RD RI was 3 at initial treatment a partial response was defined as a 1-point decline after treatment; and (3) no change (NC), in which there was no significant improvement in affected organs and clinical symptoms and change of IgG4-RD RI <2 points (8, 9, 15).

Relapse was defined as the recurrence of worsened/new disease manifestations or abnormality of organ-specific imaging findings despite treatment, with or without elevated serum IgG4 (8).

### Statistical Analysis

Continuous variables are presented as mean ± standard deviation (SD), median, or interquartile range (IQR), and compared by *t*-test, whereas categorical variables were compared by the chi-square test, Fisher Exact test, or Mann–Whitney test. The Kaplan–Meier curves and log-rank test were used to calculate the cumulative relapse rate. Statistical analyses were performed using IBM SPSS statistics (Version 22.0, IBM, Armonk, NY, USA).

## Results

### Demographic and Baseline Features

From January 2012 to July 2018, 155 IgG4-RD patients who conformed to the inclusion criteria were selected. Among them, 90 were treated with GCs plus CYC (group I) and 65 were treated with GCs plus MMF (group II). Compared to group I, patients in group II had more paranasal sinus (35.38 vs. 20.00%, *p* < 0.05), pancreas (64.62 vs. 33.33%, *p* < 0.01), and bile duct (35.38 vs. 18.89%, *p* < 0.05) involvement.

To minimize the influence of confounders on the results of the study, we used 1:1 propensity score-matched (PSM) analysis to match group I patients with group II patients based on gender, age, involvement organs (meninges, pituitary gland, lacrimal glands, parotid glands, submandibular glands, nasal cavity lesions, thyroid, lungs, lymph nodes, aorta/large blood vessels, heart/pericardium, retroperitoneal fibrosis, sclerosing mediastinitis, sclerosing mesenteritis, pancreas, liver, bile ducts, kidney, and skin), and other involvement and constitutional symptoms not attributable to the involvement of a particular organ (weight loss, fever, and fatigue caused by active IgG4-RD). At the end of the analysis, 108 patients were selected (54 in each group), of whom 49 were diagnosed with definite IgG4-RD (group I vs. group II, 24 vs. 25), 8 with probable IgG4-RD (group I vs. group II, 5 vs. 3), and 51 with possible IgG4-RD (group I vs. group II, 25 vs. 26). In the post-match model, the differences of baseline characteristic between group I and group II was not statistically significance. All patients were treated with prednisone, which was gradually tapered down according to the individual's clinical response. The analyses indicated that the initial prednisone dosages were similar between the two groups (41.13 vs. 41.33 mg/day, respectively; *p* = 0.54). In group I, patients received CYC at a mean initial dose of 54.75 mg/day (oral, 50–100 mg/day). They maintained the initial dose for 3 months, after which it was reduced to 50 mg per day or every other day, consistent with previous treatment regimens (9). The mean cumulative dose of CYC over 12 months was 11.30 g. Patients in group II received MMF at a mean initial dose of 1,060 mg/day (1000–1500 mg/day), which was maintained for 6 months and decreased to 500–1000 mg/day for 6 months, consistent with previous reports (8, 16, 17). The baseline characteristics of the two groups before and after matching are summarized in Table 1.

**Table 1:** Baseline characteristics before and after matching on the propensity score.

**Variables**	**Before matching**		***p*-value**	**After matching**		***p*-value**
	**Group I (*N* = 90)**	**Group II (*N* = 65)**		**Group I (*N* = 54)**	**Group II (*N* = 54)**	
Male, *N* (%)	68 (75.56%)	42 (64.62%)	0.14	37 (68.52%)	37 (68.52%)	1.00
Age at onset (years)	57.50 (52.00–63.75)	55.00 (48.00–62.00)	0.11	56.50 (9.94)	53.96 (12.22)	0.24
Allergy, *N* (%)	43 (47.78%)	34 (52.31%)	0.58	23 (42.59%)	29 (53.70%)	0.25
Biopsy, *N* (%)	46 (51.11%)	36 (55.38%)	0.60	31 (57.41%)	30 (55.56%)	0.44
Imaging examination						
CT scan	61 (67.78%)	48 (73.85%)	0.41	36 (66.67%)	42 (77.78%)	0.20
MRI	23 (25.56%)	18 (27.69%)	0.77	12 (22.22%)	12 (22.22%)	1.00
PET	28 (31.11%)	15 (23.08%)	0.27	19 (35.19%)	14 (25.93%)	0.30
Others	19 (21.11%)	18 (27.59%)	0.34	11 (20.37%)	14 (25.93%)	0.49
Definite IgG4-RD, *N* (%)	35 (38.89%)	30 (46.15%)	0.37	24 (44.44%)	25 (46.30%)	0.85
Probable IgG4-RD, *N* (%)	8 (8.89%)	4 (6.15%)	0.76	5 (9.26%)	3 (5.55%)	0.72
Possible IgG4-RD, *N* (%)	47 (52.22%)	31 (47.70%)	0.58	25 (46.30%)	26 (48.15)	0.85
Number of organs	4 (2–6)	4 (3–5.5)	0.13	4.00(2.75–6.00)	4.5 (3.00–6.00)	0.23
Multiorgan disease (≥3 organs), *N* (%)	46 (51.11%)	42 (64.62%)	0.09	30 (55.56%)	37 (68.52%)	0.17
Disease duration (months)	8.50 (3.00–36.00)	12.00 (5.00–36.00)	0.15	6.50 (3.00–24.00)	12.00 (2.00–36.00)	0.23
Organ involvements, *N* (%)						
Meninges	3 (3.33%)	0	0.27	0	0	—
Pituitary glands	3 (3.33%)	2 (3.08%)	1.00	1 (1.85%)	1 (1.85%)	1.00
Orbital lesion	6 (6.67%)	4 (6.15%)	0.90	4 (7.41%)	4 (7.41%)	1.00
Lacrimal glands	32 (35.56%)	25 (38.56%)	0.71	18 (33.33%)	22 (40.74%)	0.43
Parotid glands	20 (22.22%)	10 (15.38%)	0.29	10 (18.52%)	9 (16.67%)	0.80
Submandibular glands	37 (41.11%)	32 (49.23%)	0.32	24 (44.44%)	28 (51.85%)	0.44
Nasal cavity lesions and sinusitis	18 (20.00%)	23 (35.38%)	<0.05	12 (22.22%)	21 (38.89%)	0.06
Thyroid	3 (3.33%)	2 (3.08%)	1.00	2 (3.70%)	1 (1.85%)	1.00
Lung	32 (35.56%)	24 (36.92%)	0.86	22 (40.74%)	22 (40.74%)	1.00
Lymph nodes	34 (37.78%)	30 (46.15%)	0.30	23 (42.59%)	28 (51.85%)	0.34
Aorta and large blood vessels	18 (20.00%)	7 (10.77%)	0.12	10 (18.52%)	7 (12.96%)	0.43
Heart/pericardium	2 (2.22%)	2 (3.08%)	1.00	1 (1.85%)	2 (3.70%)	1.00
Retroperitoneal fibrosis	36 (40.00%)	13 (20.00%)	0.08	16 (29.63%)	13 (24.07%)	0.52
Sclerosing mediastinitis	10 (11.11%)	6 (9.23%)	0.70	7 (12.96%)	6 (11.11%)	0.77
Sclerosing mesenteritis	0	1(1.54%)	0.42	3 (5.56%)	2 (3.70%)	1.00
Pancreas	30 (33.33%)	42 (64.62%)	<0.01	27 (50.00%)	32 (59.26%)	0.33
Liver	3 (3.33%)	2 (3.08%)	1.00	2 (3.70%)	1 (1.85%)	1.00
Bile ducts	17 (18.89%)	23 (35.38%)	<0.05	16 (29.63%)	18 (33.33%)	0.68
Skin	5 (5.56%)	2 (3.08%)	0.70	4 (7.41%)	2 (3.70%)	0.68
Kidney	13 (13.33%)	7 (10.77%)	0.50	9 (16.67%)	7 (12.96%)	0.59
Prostate	17 (18.89%)	9 (13.85%)	0.41	8 (14.81%)	9 (16.67%)	0.79
Constitutional symptoms not attributable to involvement of a particular organ^a^	10 (11.11%)	4 (6.15%)	0.29	4 (7.41%)	4 (7.41%)	1.00
Other involvement: specify^b^	24 (26.67%)	18 (27.69%)	0.89	17 (31.48%)	11 (20.37%)	0.19
Serological						
Eosinophils (10^9^/L)	2.60 (0.70–5.80)	2.50 (0.70–5.20)	0.69	3.2 (1.60–5.60)	2.45 (0.53–5.83)	0.23
C-reactive protein (mg/L)	3.51 (0.81–9.43)	3.53 (1.26–11.26)	0.66	4.40 (1.73–13.88)	2.48 (0.79–8.25)	0.13
Erythrocyte sedimentation rate (mm/h)	32.50 (9.75–61.25)	23.00 (11.75–67.25)	0.73	32.00 (13.00–65.00)	24.50 (8.25–60.50)	0.43
IgE (KU/L)	180.00 (97.23–780.00)	263.00 (78.73–631.75)	0.18	327.00 (97.23–780.00)	206.00 (102.00–644.50)	0.57
IgG4 (mg/L)	5610.00 (2252.50–19,125.00)	11,000.00 (4190.00–19,300.00)	0.12	11,000 (4183.50–20,550.00)	7510 (2925.00–20,950.00)	0.59
IgG4-RD RI	11.63 ± 6.16	11.72 ± 5.06	0.70	12.28 ± 5.87	12.50 ± 4.90	0.65
Initial GC dose (mg/day)	42.69 ± 10.32	41.16 ± 8.37	0.18	41.13 ± 8.09	41.33 ± 10.89	0.54

a *Weight loss, fever, and fatigue caused by active IgG4-RD*.

b *Prostate, breast, gallbladder involvement, and others*.

### Treatment Response

**Figure 1:**
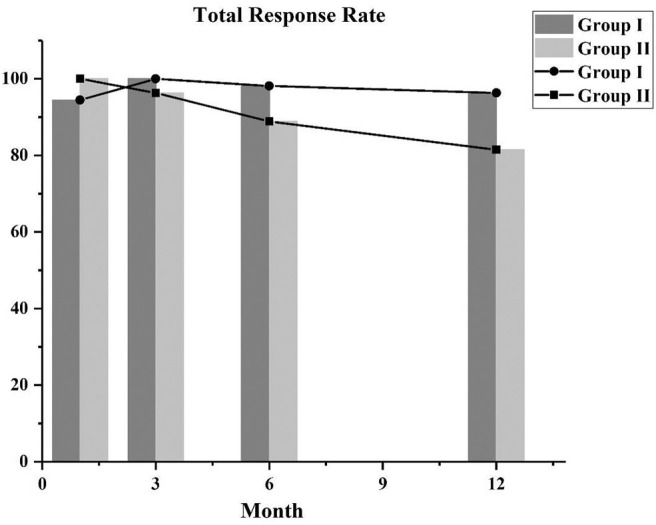
The total response rates of two groups at 1, 3, 6, and 12 months.

After using the PSM approach to control covariates, we compared the response rates of the two groups. The total response rates of the patients in the two groups are shown in Figure 1, and the majority of the patients achieved CR or PR. In the first month follow-up period, in group I, 46 patients achieved PR, 5 attained CR, and 3 did not respond to treatment (NC), and in group II, 2 attained CR and 52 achieved PR. After follow-up at 3 months, the 3 NC patients in group I achieved PR. In the third month follow-up period, the majority of patients in group I (10 achieved CR and 44 achieved PR) and group II (7 achieved CR and 45 achieved PR) had a good response. At the 6- and 12-month follow-up, the total response rates in group I were higher than those in group II, but there was no statistical difference between the two groups (Table 2).

**Table 2:** Response rates of group I and group II.

**Period (months)**	**Status, *N* (%)**	**Group I (*N* = 54)**	**Group II (*N* = 54)**
1	CR	5 (9.26%)	2 (3.70%)
	PR	46 (85.19%)	52 (96.30%)
	NC	3 (5.56%)	0
	Relapse	0	0
3	CR	10 (18.52%)	7 (12.96%)
	PR	44 (81.48%)	45 (83.33%)
	NC	0	0
	Relapse	0	2 (3.70%)
6	CR	18 (33.33%)	17 (31.48%)
	PR	35 (64.82%)	33 (61.11%)
	NC	0	0
	Relapse	1 (1.85%)	4 (7.41%)
12	CR	30 (55.56%)	27 (50.00%)
	PR	23 (42.59%)	25 (46.30%)
	NC	0	0
	Relapse	1 (1.85%)	2 (3.70%)

*CR, complete response; NC, no change; PR, partial response*.

**Figure 2:**
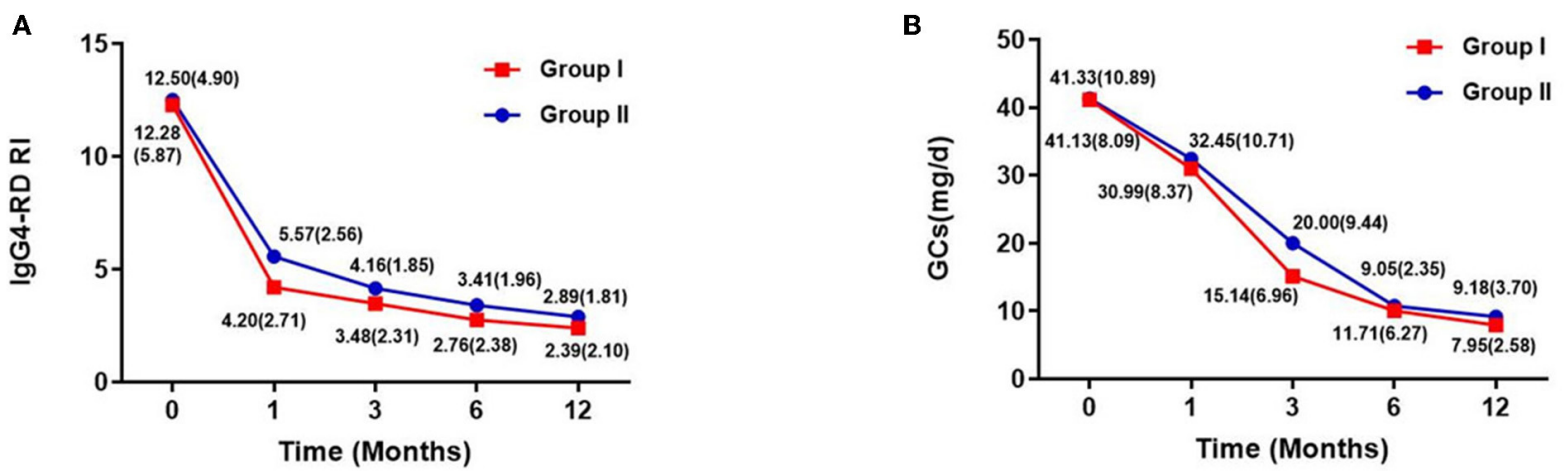
IgG4-RD RI [mean (SD)] and GCs dose [mean (SD)] of two groups within follow-up time. **(A)** IgG4-RD RI evaluated during the follow-up period. **(B)** GC doses of each patient at baseline, and at 1, 3, 6, and 12 months. Doses of glucocorticoids are presented as means.

The IgG4-RD RI and GC dose at each follow-up period are shown in Figures 2A,B. They both decreased over time, especially during the first 6 months of treatment, after which they stabilized. At the last follow-up period, IgG4-RD RI significantly declined in both groups, and the mean (SD) IgG4-RD RI decreased to 2.39 (2.10) in group I and 2.89 (1.76) in group II, with a mean decline of 80.54 and 76.88% of baseline level, respectively. Also, the GCs dose was reduced from the initial 41.13 to 7.95 mg/day in group I, and from 41.33 to 9.18 mg/day in group II, with no significant difference between both groups.

### Disease Relapse

**Figure 3:**
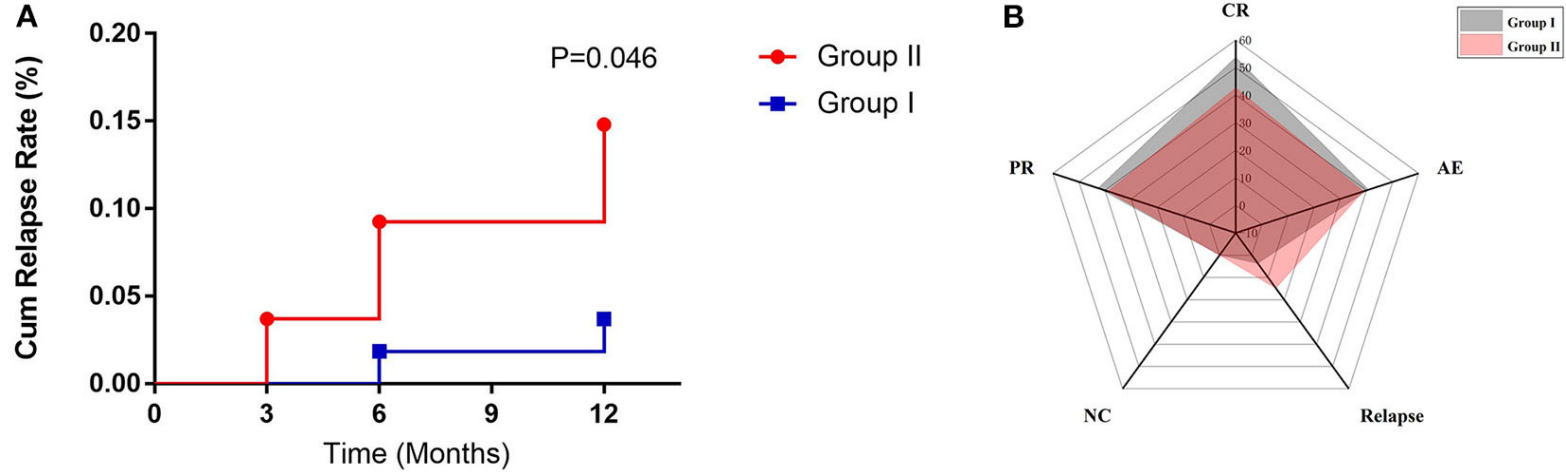
**(A)** The relapse rate of two groups within follow-up time. **(B)** Radar map for evaluating the efficacy of two treatment regimens by response rate (CR, PR, and NC), relapse rate (RR), and adverse effect (AE).

In group I, 2 patients suffered relapse; one had a recurrence in the pancreas in the 6th month, which manifested as abdominal pain and the other had a worsened lung image on the 12th month with cough. In group II, 8 patients suffered relapse; 2 had recurrence on the skin, lung, and lacrimal glands at the 3rd month visit, 4 relapsed in the 6th month (lacrimal glands and pancreas), and 2 had recurrence in the 12th month (lacrimal glands and paranasal sinus). In group II, recurrence at the paranasal sinus, lacrimal glands, skin, lung, bile ducts, and pancreas accounted for 25, 50, 12.5, 12.5, 12.5, and 25%, respectively, with the lacrimal glands being the most vulnerable to recurrence. The cumulative recurrence rate in groups I and II was 3.7 and 14.8%, respectively, and group II had a higher relapse rate than group I (*p* = 0.046) (Figure 3A).

The follow-up data after retreatment in these 10 patients showed that they all achieved remission after the immunosuppressants were changes and/or the dose of GCs was increased according to their condition. In group I, for one patient, we increased the GCs dosage and replaced CYC with MMF, whereas for the second patient, the GCs dosage was increased. Of the 8 relapsed patients in group II, the treatment was converted to GCs and CYC for 3, iguratimod was added for 1, GC dose was increased and MMF was replaced with MTX for 1, and previous regimens with increased dosages were maintained in 3 patients. The details of relapsed patients in group I and group II are shown in Table 3.

**Table 3:** Relapsed patients in Group I and Group II.

**Patients**	**Group**	**Involvement organ**	**Relapsed organ**	**Time of relapse**	**Added treatment**	**Outcome**
1	I	Aorta, retroperitoneal fibrosis, and pancreas	Pancreas	6	Increased GC dosage, changed CYC to MMF	PR
2	I	Submandibular glands, lymph nodes, retroperitoneal fibrosis, pancreas, and bile ducts	Lung	12	Increased GC dosage	PR
3	II	Aorta, retroperitoneal fibrosis, pancreas, and bile ducts	Skin and lung	3	GCs, MMF, and iguratimod	PR
4	II	Lacrimal glands, aorta, lymph nodes, lung, pancreas, and bile ducts	Lacrimal glands	3	Increased GC dosage, changed MMF to MTX	PR
5	II	Submandibular glands, lymph nodes, pancreas, lacrimal glands, and lung	Lacrimal glands	6	Increased GC and MMF dosage	PR
6	II	Submandibular glands, lymph nodes, pancreas, lacrimal glands, prostate, and lung	Lacrimal glands	6	Increased GC dosage and changed MMF to CYC	CR
7	II	Pancreas and bile ducts	Pancreas and bile ducts	6	Changed MMF to CYC	PR
8	II	Aorta, retroperitoneal fibrosis, and pancreas	Pancreas	6	Increased GC dosage	PR
9	II	Lacrimal gland, lung, and paranasal sinus	Lacrimal gland and paranasal sinus	12	Changed MMF to CYC	CR
10	II	Parotid glands and paranasal sinus	Paranasal sinus	12	Increased GC and MMF dosage	PR

*CR, complete response; CYC, Cyclophosphamide; GC, glucocorticoid; MMF, Mycophenolate mofetil*.

*MTX, methotrexate; PR, partial response*.

### Adverse Effects

Table 4 shows the adverse effect (AEs) of therapy, with infections as the main adverse reaction caused by the two treatment regimens. In addition, a patient in group I developed persistent platelet reduction after treatment with GCs plus CYC, so CYC was replaced with MMF and the patient's platelet count was monitored. It was observed that the patient's platelet counts gradually returned to normal after CYC was replaced. Also, a 37-year-old female patient's treatment plan was changed to oral GCs plus MMF because she had amenorrhea and a gastrointestinal reaction after CYC treatment. For another patient in group I, CYC was replaced with MMF due to liver damage manifested as elevated transaminase after using CYC. In group II, MMF was discontinued in three patients or replaced with CYC due to recurrent infections. Other adverse reactions in group II were similar to those in group I, the majority of which were infections and gastrointestinal reactions. There was no significant difference in treatment-related side effects in the two groups.

**Table 4:** Adverse events in response to glucocorticoid or immunosuppressant observed during treatment in two groups.

**Adverse events**	**Number of patients**		***p*-Value**
	**Group I**	**Group II**	(^*^ < 0.05)
Infection			
Upper respiratory tract infection	5	7	0.54
Pneumonia	0	2	0.50
Herpes zoster	2	1	1.00
Other	2	1	1.00
Glucose intolerance			
Newly diagnosed with diabetes mellitus	2	1	1.00
Aggravation of diabetes mellitus	2	3	1.00
Gastrointestinal reaction	6	5	0.75
Hematological system	1	0	1.00
Liver damage	1	0	1.00
Hemorrhagic cystitis	0	0	—
Other adverse reaction	0	0	—

Based on comprehensive assessment of disease response, relapse rate, and adverse reactions, it was suggested that GCs plus CYC had a better therapeutic effect than GCs plus MMF (Figure 3B).

## Discussion

Several immunosuppressants have been used as a steroid-sparing treatment for IgG4-RD, but there has been no head-to-head study comparing the efficacies between steroid-sparing drugs. In this retrospective cohort study, we aimed to explore the efficacies of GCs plus MMF therapy and GCs plus CTX and compare them in order to determine a better therapeutic strategy for IgG4-RD patients and prevent disease recurrence.

In this study, CYC and MMF were used because their efficacies in IgG4-RD had been proven in previous studies. In a recent study, CYC plus GCs yielded a lower relapse rate and higher response rate relative to GC monotherapy. Similar to the results of combined CYC regimens, a study from India showed clinical improvement in patients who were treated with GCs plus MMF, with no case of relapse within a median duration of 8 months. Furthermore, an RCT study from China reported that GCs plus MMF was more effective than GCs monotherapy.

In our study, IgG4-RD patients responded well to GCs plus CYC or MMF, and a majority of them experienced marked improvements within a month of treatment, which were manifested as improvement in the affected organs; reductions in the concentrations of serum ESR, CRP, IgG, and IgG4; as well as successful GC tapering. Both combined treatment regimens were efficient for IgG4-RD patients, having almost the same response rate during a year follow-up period. Comparing the ability of the two immunosuppressants to prevent flare by relapse rate, our results demonstrated that 10 patients (2 with CYC and 8 with MMF) relapsed, and relapse occurred earlier and more frequently in those receiving MMF than CYC (14.8 vs. 3.7%). Additionally, patients receiving CYC had less cumulative GCs than those receiving MMF, indicating that the steroid-sparing effect of CYC is superior to that of MMF. Therefore, the combined treatment with CYC decreased organ recurrence and maintained disease remission much better than combined treatment with MMF. Therefore, it was suggested that a low-dose CYC combination treatment regimen might be a better choice than low-dose MMF therapy in IgG4-RD patients with multiple organ involvement.

In a recent RCT study that enrolled IgG4-RD patients with multiple organ involvement, it was pointed out that lacrimal glands and paranasal sinus were most susceptible to recurrence in patients receiving low-dose MMF plus GCs (8). However, in a prospective cohort study of combined CYC treatment for IgG4-RD, it was observed that no patient experienced recurrence of superficial organs such as lacrimal glands and paranasal sinuses in the group receiving GCs plus CYC (9). In the present study, the relapse of patients in group II was more likely due to recurrence in the lacrimal glands (4/8, 50.00%) and paranasal sinus (2/8, 25.00%), and the other relapsed organs including the pancreas and lung were similar to those in group I. In accordance with the results obtained from this study, it is necessary to find other more efficient treatments for lacrimal glands and paranasal sinus involvement.

Regarding safety issues, since low doses of CYC and MMF were applied in this study, both drugs were well tolerated, and this is consistent with previous IgG4-RD studies. The main adverse reactions in the CYC or MMF groups were infections and gastrointestinal reactions, and they did not differ between both groups (Table 4). Previous studies observed that the incidence of severe infection significantly increased in the Asian population compared with those of other races when MMF at 3 g/day was used in the treatment of lupus nephritis (16, 17). Based on our clinical experience, patients over 60 years of age showed vulnerability to MMF of more than 1.5 g per day, with an increased risk of opportunistic infection, while most IgG4-RD patients are older than 60 years old. In the CYC group, two patients had drug-induced liver damage and persistent reduction in platelets, respectively, but the symptoms disappeared after CYC was discontinued. When CYC is used, it is applied only in patients with internal organ involvement. We carefully monitored short-term and long-term side effects of CYC, and strictly controlled the cumulative dose for every patient, although in our experience in treating systemic rheumatic diseases, such as systemic lupus erythematosus and systemic vasculitis, Chinese patients tolerate CYC much better than patients reported from Western countries.

So far, this is the first study to compare the effects of different immunosuppressive agents in IgG4-RD, although there are some limitations. Since the study is a retrospective cohort study, unknown variables might affect the results, leaving room for potential bias such as adverse effects that were as retrieved from the patients' hospital records. However, the results are quite reliable because there were no statistically significant differences in the baseline characteristics between the two groups.

In conclusion, most IgG4-RD patients had a good response to both treatment regimens, but GCs plus CYC had a lower rate of relapse within a year than GCs plus MMF. This suggests that GCs plus CYC might be a more effective treatment option for IgG4-RD patients with internal organ involvement. However, larger prospective and RCT studies are required to understand the role of CYC and MMF in IgG4-RD.

## Data Availability Statement

All datasets generated for this study are included in the article/supplementary material.

## Author Contributions

XL and YP designed the research, analyzed data, and wrote the manuscript. PZ, ZL, JL, and HL collected and analyzed data and revised the manuscript. XZh, FZ, and XZe optimized the research and proofread the paper. YF and WZ designed the study and wrote the manuscript. All authors contributed to the article and approved the submitted version.

## Conflict of Interest

The authors declare that the research was conducted in the absence of any commercial or financial relationships that could be construed as a potential conflict of interest.
